# Ginsenoside Rg3 Mitigates Atherosclerosis Progression in Diabetic apoE–/– Mice by Skewing Macrophages to the M2 Phenotype

**DOI:** 10.3389/fphar.2018.00464

**Published:** 2018-05-09

**Authors:** Mengqi Guo, Jie Xiao, Xi Sheng, Xinyu Zhang, Yuanyuan Tie, Lei Wang, Lang Zhao, Xiaoping Ji

**Affiliations:** Key Laboratory of Cardiovascular Remodeling and Function Research, Chinese Ministry of Education and Chinese Ministry of Health, Department of Cardiology, Qilu Hospital of Shandong University, Jinan, China

**Keywords:** Ginsenoside Rg3, PPARγ, M2 macrophages, atherosclerosis, diabetes

## Abstract

Atherosclerosis (AS) in diabetic patients is often associated with low stability, which might be largely attributed to unfavorable macrophage polarization and increased inflammatory response induced by hyperglycaemia. Ginsenoside Rg3 is one of the main active principles of Panax Ginseng, which has been reported to be a natural ligand of peroxisome proliferator-activated receptor-gamma (PPARγ), a key nuclear transcriptional factor involved in inflammation and macrophage differentiation. However, it remains unclear if Rg3 could exert protective effects on plaque stability in diabetes. In this study, we investigated the role of ginsenoside 20(S)-Rg3 in macrophage polarization and AS plaque stability using advanced glycation end products-treated macrophages and diabetic AS mice models. *In vitro*, advanced glycation end products (AGEs) treatment promoted the expression of proinflammatory molecules and M1 surface markers, whereas 20(S)-Rg3 could reverse the M1 polarization to the M2 phenotype. *In vivo*, the administration of 20(S)-Rg3 promoted AS lesion stability and reduced the plaque burden, accompanied by increased M2 macrophages and reduced M1 macrophages. In addition, PPARγ antagonist GW9662 co-administration mostly blocked these effects, suggesting the important role of PPARγ pathways in mediating 20(S)-Rg3 effects in macrophage polarization and atherosclerosis progression. Together, these results demonstrated an immunomodulatory role of ginsenoside 20(S)-Rg3 in promoting macrophages to a profile of the M2 type through PPARγ-dependent mechanisms, and indicated a potential role of 20(S)-Rg3 in the prevention and treatment of diabetic atherosclerosis.

## Introduction

Atherosclerosis is a multifactorial progressive inflammatory disease of the arterial wall and is the underlying basis of many other cardiovascular diseases (CVDs) such as myocardial infarction and stroke (Viola and Soehnlein, 2015). Macrophages play a crucial role in the genesis and progression of atherosclerosis (Ley et al., 2011; Zeller and Srivastava, 2014). As highly plastic and versatile cells, macrophages can grossly differentiate into classically (M1) or alternatively (M2) activated macrophages. The M1 macrophage produces various proinflammatory cytokines, such as interleukin-6 (IL-6) and tumor necrosis factor-α (TNF-α), inducing pro-inflammatory effects. Conversely, M2 macrophages can secrete high levels of anti-inflammatory cytokines, such as interleukin-10 (IL-10) and transforming growth factor (TGF-β) (Biswas et al., 2012; Labonte et al., 2014). Both M1 and M2 macrophages are found in atherosclerosis plaque. Pro-inflammatory M1 macrophages are dominant in more advanced plaque lesions, while anti-inflammatory M2 macrophages are dominant in early-stage plaques and exhibit atheroprotective effects (Khallou-Laschet et al., 2010). Therefore, promoting favorable M1/M2 macrophage polarization might be a new approach to alleviate inflammation and increase plaque stability.

Diabetes is a complex metabolic disease that is characterized by hyperglycemia arising from deficiencies in insulin action. In type 1 diabetes (T1DM), a direct deficiency of insulin results from an autoimmune-driven destruction of the pancreatic beta cells. In type 2 diabetes (T2DM), there is an impaired response of peripheral tissues to insulin, known as insulin resistance, which promotes hyperinsulinemia and eventual beta cell dysfunction. Despite their different etiologies, both type 1 and type 2 diabetes are associated with the accelerated development of atherosclerotic lesions as well as an increased incidence of CVDs (Zeadin et al., 2013). Prolonged exposure to hyperglycemia is now recognized as a major factor in the pathogenesis of diabetic complications, including atherosclerosis (Aronson, 2008; Funk et al., 2012; Kanter and Bornfeldt, 2013). In hyperglycaemic conditions, AGEs accumulate and the receptors for AGE (RAGE) are activated (Yue et al., 2015). Binding of AGEs to their receptor RAGE could initiate cellular signals that activate NF-κB, which results in the transcription of proinflammatory factors (Lin, 2006). Previous studies found that AGEs could enhance macrophage polarization into the M1 phenotype, mediating inflammation and atherosclerosis (Jin et al., 2015; Zhou et al., 2016). Therefore, we speculate that blocking or even reversing the effects of AGEs on macrophage polarization might be a good strategy to alleviate atherosclerosis progression and increase plaque stability in diabetes.

Ginseng, the root of Panax ginseng C. A. Meyer, has been consumed as an herbal drug in traditional oriental medicine for preventive and therapeutic purposes for more than 2000 years. Ginsenoside Rg3 (GRg3), one of the major active saponins isolated from ginseng, has been demonstrated to exert beneficial effects on diabetes and CVDs (Kim et al., 2015; Wang et al., 2015). Recent studies have reported that ginsenoside Rg3 can act as a natural ligand of peroxisome proliferator-activated receptor-gamma (PPARγ) (Kwok et al., 2012), a key nuclear transcriptional factor involved in inflammation and macrophage differentiation (Odegaard et al., 2007; Zhang et al., 2016). Ginsenosides 20(S)-Rg3 and 20(R)- Rg3 are an enantiomeric pair that differ in the spatial orientation of the hydroxyl group on the chiral center at carbon-20 (C20), and the PPARγ agonist activity of 20(S)-Rg3 is much stronger than that of 20(R)-Rg3 (Kwok et al., 2012). However, the effect of 20(S)-Rg3 on macrophage polarization and diabetic atherosclerosis has not yet been extensively investigated. The present study tests if 20(S)-Rg3 could alleviate inflammation and atherosclerosis by inhibiting AGEs-induced classical activation of M1 macrophages and promoting M2 polarization in diabetic conditions.

## Materials and Methods

### Cell Culture and Treatment

The bone marrow cells (BMDMs) were isolated from female C57BL/6 mice by flushing the femur and tibia with PBS. BMDMs were cultured at 37°C with 5% CO2 in Dulbecco’s modified Eagle’s medium (DMEM; Gibco, United States) supplemented with 10% fetal bovine serum (FBS; Gibco, United States), 20 ng/ml macrophage colony-stimulating factor (M-CSF; Peprotech, United Kingdom), 1% glutamine (Life Technologies, United States), and 1% penicillin/streptomycin (Beyotime, China). Cells were harvested on day 7 for experiments *in vitro*. A human acute monocytic leukemia cell line (THP-1) was obtained from the Cell Bank of the Chinese Academy of Sciences. Cells were cultured in RPMI 1640 medium (Gibco, United States) supplemented with 10% FBS (Gibco, United States). To induce differentiation of macrophages, THP-1 cells were cultured in the presence of 100 ng/mL phorbol-12-myristate-13 acetate (PMA; Sigma, United States) for 24 h.

Macrophages were pretreated with 20(S)-Rg3 (25 μM, dissolved in 0.1% DMSO; Fleton, China) in the presence or absence of GW9662 (3 μM; Selleck, United States) for 1 h, and then stimulated with AGEs (200 μg/ml; Biovision, United States) for 24 h. An equal volume of DMSO was added to the controls.

### Cell Viability Assay

Cells were seeded into 96-well plates for 24 h, and proliferation was determined using the Cell Counting Kit-8 (CCK-8; Dojindo, Japan) according to the manufacturer’s instructions. Optical density was measured at 450 nm.

### Western Blot Analysis

Proteins were separated by 10% sodium dodecyl sulfate-polyacrylamide gel electrophoresis (SDS–PAGE) and transferred onto polyvinylidene difluoride (PVDF) membranes (Bio-Rad, China). Membranes were blocked with 5% non-fat dry milk in TBST at room temperature for 2 h and incubated overnight at 4°C with primary antibodies against PPARγ (1:1000, ab59256; Abcam), inducible nitric oxide synthase (iNOS) (1:1000, ab129372; Abcam), arginase-1 (Arg-1) (1:1000, ab133543; Abcam) respectively and β-actin (1:1000, 2SGB-BIO, TA-09). Membranes were visualized with the chemiluminescence reagent, and the intensities of protein bands were evaluated by ImageJ (National Institutes of Health, United States). Protein expression was assessed relative to β-actin.

### Flow Cytometry

RAW264.7 cells (a mouse macrophage cell line) and THP-1 cells were pretreated with 20(S)-Rg3 (25 μM) in the presence or absence of GW9662 (3 μM) for 1 h, and then stimulated with AGEs (200 μg/ml) for 24 h. Cells were washed, blocked and then incubated for 30 min with APC-conjugated CD206 (17-2061-82 and 17-2069-42 eBioscience, for RAW264.7 cells and THP-1 cells, respectively) and PE-conjugated CD86 (12-0862-82 and 12-0869-42 eBioscience, for RAW264.7 cells and THP-1 cells, respectively) using the appropriate isotype controls. The results were acquired using a BD FACS Caliber flow cytometer (BD Biosciences, United States) and analyzed with FlowJo v9.0 software (Tree Star, Inc., FlowJo v9.0, Ashland, OR, United States).

### Enzyme-Linked Immunosorbent Assays (ELISA)

The concentration of TNF-α, IL-6, IL-10, and TGF-β in culture supernatants of BMDMs were measured by ELISA kits (Proteintech, China) following the manufacturer’s protocol.

### Animal Model

Eight-week-old male ApoE-/- mice (*n* = 40) were randomly divided into the following 4 groups (*n* = 10 per group): non-diabetic control group, DM (diabetic mellitus) + placebo group, DM +20(S)-Rg3 group, and DM +20(S)-Rg3 +GW9662 group. Diabetes was initiated by the administration of 5 daily intraperitoneal injections of 50 mg/kg streptozotocin (STZ) in citrate buffer (0.05 mol/L; pH 4.5). Mice with continuous blood glucose levels of >300 mg/dL were considered diabetic and were included in the DM cohorts. Mice received normal chow for the remaining 12 weeks. During the 8–12th weeks, mice were given Rg3 at a dose of 10 mg/kg i.p. once every 2 days (Tian et al., 2016) with oral gavage of GW9662 at 3 mg/kg per day (Xuan et al., 2017). The experimental protocol complied with the Animal Management Rules of the Chinese Ministry of Health (documentation 55, 2001) and was approved by the Animal Ethics Committee of Shandong University.

### Histology and Immunohistochemistry

To assess the overall burden and distribution of atherosclerosis, en face lesion staining with Oil-Red O was performed as previously described (Zhang C. et al., 2010). Cross-sections of the aortic roots (predilection site of atherosclerosis) were stained with haematoxylin and eosin (H&E) following a standard protocol of our lab.

The content of lipids of aortic plaques was detected by Oil-Red O staining, and the collagen content was assessed with Sirius Red-stained slides under polarizing light (Gordon and Martinez, 2010). The immunohistochemical staining was performed as previously described (Zhang C. et al., 2010). Targeted proteins were identified by antibodies against alpha smooth muscle actin antibody (α-SMA) (1:200, ab5694; Abcam) and monocyte/ macrophage antigen [MOMA-2] (1:200, MCA519G; AbD). Histological and immunohistochemical staining were analyzed using Image-Pro Plus 6.0 (IPP 6.0, Media Cybernetics, MD, United States). The plaque vulnerability index was calculated using the following formula: vulnerability index = (lipid deposit%+macrophages%)/(collagen fibers %+SMCs%) (Wen et al., 2017).

### Immunofluorescence Staining

For immunofluorescence, frozen sections were labeled with unconjugated primary antibodies against MOMA-2 (1:200, MCA519G; AbD) and a M1 marker [iNOS (1:200, ab178945; Abcam) or CD86 (1:200, NBP2-25208; Novus)], or a M2 marker [Arg1 (1:200, ab60176; Abcam) or CD206 (1:200, ab64693; Abcam)] simultaneously overnight, followed by incubation with a fluorophore-conjugated secondary antibody for 30 min. The stained sections were mounted with DAPI-containing VectorShield mounting medium (Vector) and then viewed using an Olympus BX53 fluorescence microscope.

### Statistical Analysis

All experiments were repeated at least three times, and data were presented as the mean ± S.E.M. Statistical analysis was carried out using ANOVA followed by Tukey’s *post hoc* test (GraphPad Software, United States). *P* < 0.05 was considered significant.

## Results

### 20(S)-Rg3 Induced M2 Polarization and Suppressed AGEs-Induced M1 Macrophage Activation *in Vitro*

**FIGURE 1:**
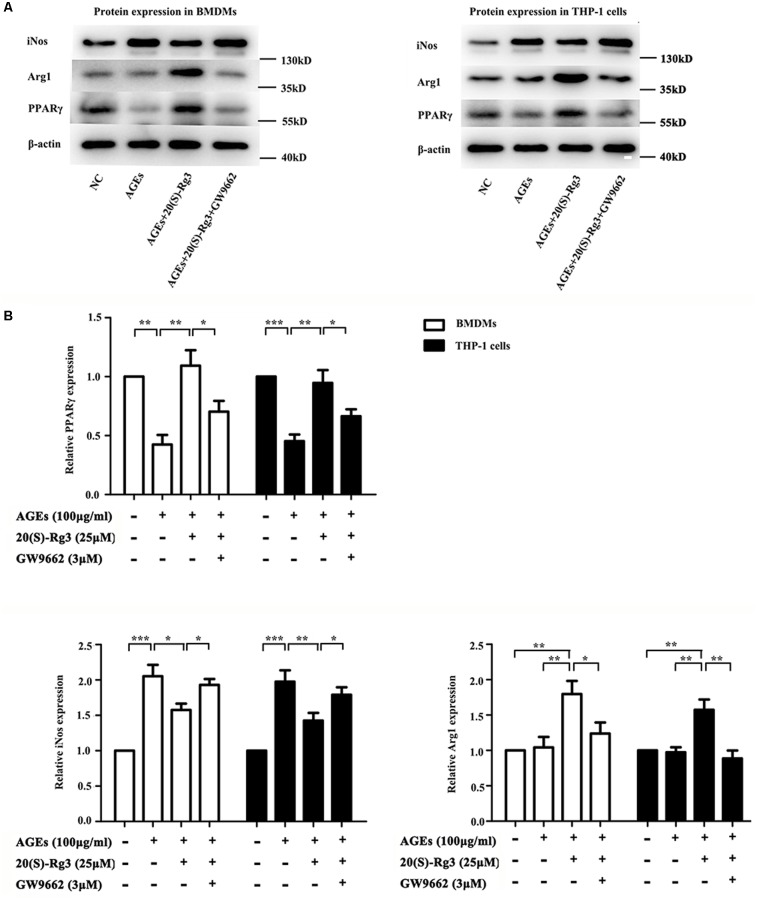
20(S)-Rg3 suppress AGEs-induced M1 macrophage activation and promote M2 polarization **(A)** Representative immunoblots of PPARγ, iNOS (M1 marker) and Arg-1 (M2 marker) in BMDMs and THP-1 cells. **(B)** Quantification of PPARγ, iNOS and Arg-1 expression relative to the β-actin level (*n* = 3 respectively for PPARγ, iNos and Arg-1 in BMDMs; *n* = 3, 3, 4 for PPARγ, iNos and Arg-1 in THP-1 cells). ^∗^*p* < 0.05, ^∗∗^*p* < 0.01, ^∗∗∗^*p* < 0.001.

**FIGURE 2:**
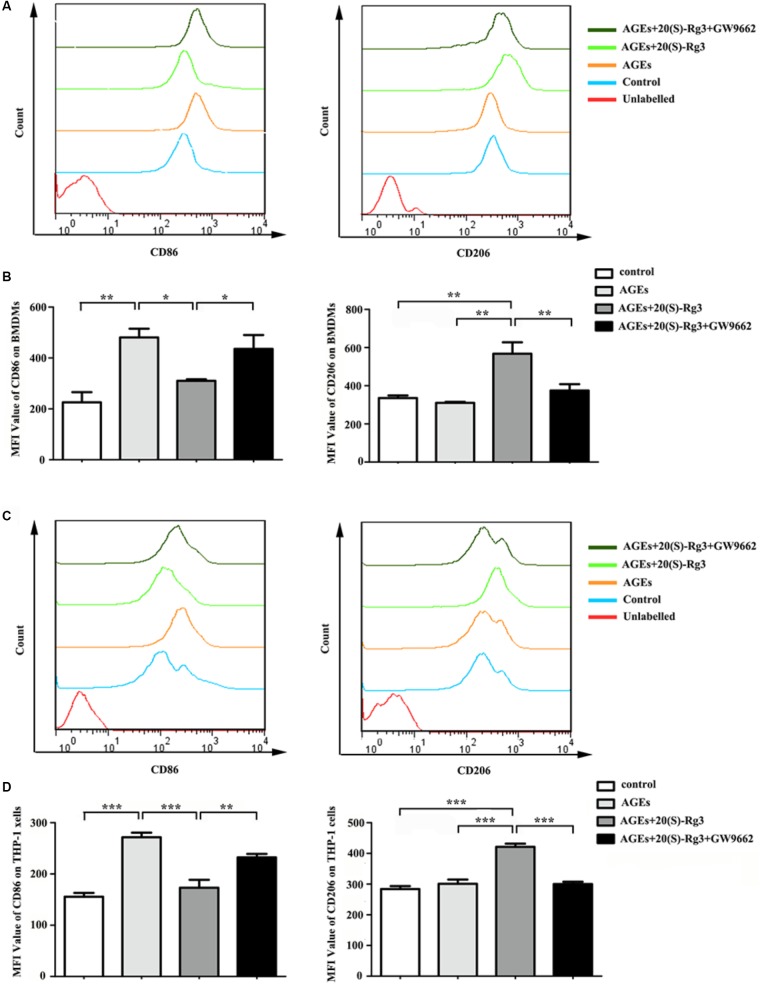
Flow cytometry analysis of M1 and M2 surface markers **(A)** The expression of CD86 (M1 surface marker) and CD206 (M2 surface marker) on RAW264.7 cells were examined by flow cytometry. **(B)** Quantification of mean fluorescence intensity (MFI) of the surface markers (*n* = 3, respectively). **(C)** The expression of CD86 and CD206 on THP-1 cells were examined by flow cytometry. **(D)** Quantification of mean fluorescence intensity (MFI) of the surface markers (*n* = 3, respectively). The results are expressed as the mean values ± S.E.M. ^∗^*p* < 0.05, ^∗∗^*p* < 0.01, ^∗∗∗^*p* < 0.001.

To determine directly if 20(S)-Rg3 could suppress AGEs-induced M1 macrophage activation and promote M2 polarization, macrophages were subjected to different treatments. As shown in **Figure 1**, PPARγ expression was downregulated by AGEs stimulation but reversed by 20(S)-Rg3 treatment in both BMDMs and THP-1 cells. AGEs treatment significantly promoted the expression of the M1 marker iNOS, but did not affect the M2 marker Arg1 expression, whereas 20(S)-Rg3 pre-incubation reduced the expression of iNOS and up-regulated that of Arg-1 (**Figure 1**). The flow cytometry analysis also revealed that AGEs treatment increased the population of M1 polarized macrophages, while pretreatment with 20(S)-Rg3 reversed the M1 polarization to the M2 phenotype (**Figure 2**). When cells were treated with the PPARγ antagonist GW9662 along with 20(S)-Rg3 and AGEs, the M2 polarizing effects of 20(S)-Rg3 were abated, suggesting the important role of the PPARγ pathways in mediating 20(S)-Rg3 effects in macrophage polarization. In addition, the treatment of macrophages with 20(S)-Rg3 at doses of 25 μM for 24 h did not show significant cytotoxicity (**Supplementary Figure S1**).

### 20(S)-Rg3 Suppressed AGEs-Induced Cytokine and Chemokine Production

**FIGURE 3:**
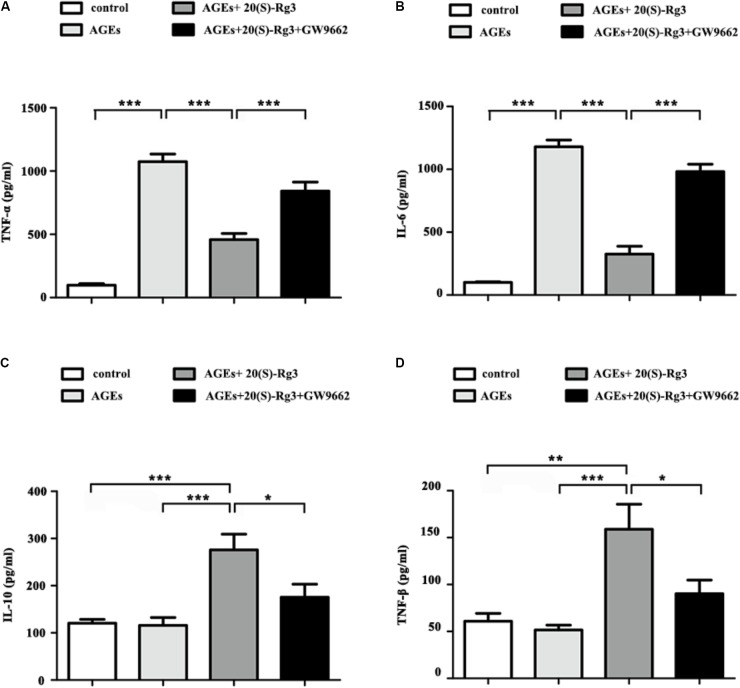
20(S)-Rg3 abated the pro-inflammatory effects of AGEs and promoted secretion of anti-inflammatory cytokines **(A–D)** ELISA for expression of pro-inflammatory cytokines (TNF-α, IL-6) and anti-inflammatory cytokines (IL-10, TGF-β) in supernatant (*n* = 4, respectively). Data are expressed as the mean values ± S.E.M. ^∗^*p* < 0.05, ^∗∗^*p* < 0.01, ^∗∗∗^*p* < 0.001.

We further assessed the role of 20(S)-Rg3 on AGEs-induced inflammatory cytokine secretion from BMDMs by ELISA. AGEs treatment significantly promoted the secretion of pro-inflammatory cytokines (IL-6, TNF-a), and slightly decreased that of anti-inflammatory cytokines (IL-10, TGF-β). Pre-incubation with 20(S)-Rg3 not only abated the pro-inflammatory effects of AGEs, but also increased the expression of anti-inflammatory molecules (**Figures 3A–D**). Pre-incubation with GW9662 significantly inhibited the anti-inflammatory effects of 20(S)-Rg3, suggesting a crucial role of PPARγ activation.

### Biochemical Parameters After Rg3 Treatment *in Vivo*

To study the ability of 20(S)-Rg3 to influence atherosclerosis progression *in vivo*, we performed an intervention study in which 20(S)-Rg3 was administered exogenously to diabetic mice. The bodyweights and biochemical parameters of the mice are described in **Table 1**. The fasting blood glucose (FBG) of the DM group was much higher than that of the control group, whereas 20(S)-Rg3 treatment significantly decreased the FBG levels. DM dramatically increased the total cholesterol (TC), low-density lipoprotein cholesterol (LDL-C) and triglyceride (TG) in the blood, whereas 20(S)-Rg3 treatment failed to significantly improve the levels of the lipids. In addition, treatment with 20(S)-Rg3 and GW9662 was well tolerated and did not impair the apparent health or survival of the mice.

**Table 1:** Biochemical parameters after 20(S)-Rg3 treatment.

Parameters	Non-diabetic apoE–/–	Diabetic apoE–/–		
		placebo	20(S)-Rg3	20(S)-Rg3 +GW9662
BW (g)	28.32 ± 1.35	25.62 ± 3.56	26.54 ± 1.78	25.24 ± 3.07
FBG (mmol/l)	6.22 ± 0.68	25.34 ± 2.55*	19.42 ± 2.77*#	23.84 ± 2.03*
TC (mmol/l)	9.06 ± 2.21	21.42 ± 5.72*	19 ± 4.15*	21.08 ± 5.14*
HDL-C (mmol/l)	1.58 ± 0.46	2.49 ± 0.56	2.3 ± 0.54	2.17 ± 0.71
LDL-C (mmol/l)	1.63 ± 0.56	4.57 ± 1.16*	3.66 ± 1.22*	4.48 ± 0.85*
TG (mmol/l)	0.75 ± 0.14	1.27 ± 0.31*	1.08 ± 0.32	1.34 ± 0.37*

*^∗^p < 0.05 vs. non-diabetic apoE–/– group; ^#^p < 0.05 vs. placebo-treated diabetic apoE–/– group. BW, body weight; FBG, fasting blood glucose; TC, total cholesterol; TG, triglycerides; HDL-C, high-density lipoprotein cholesterol; LDL-C, low-density lipoprotein cholesterol.*

### 20(S)-Rg3 Administration Reduced Plaque Burden and Enhanced Plaque Stability in Diabetic Mice

**FIGURE 4:**
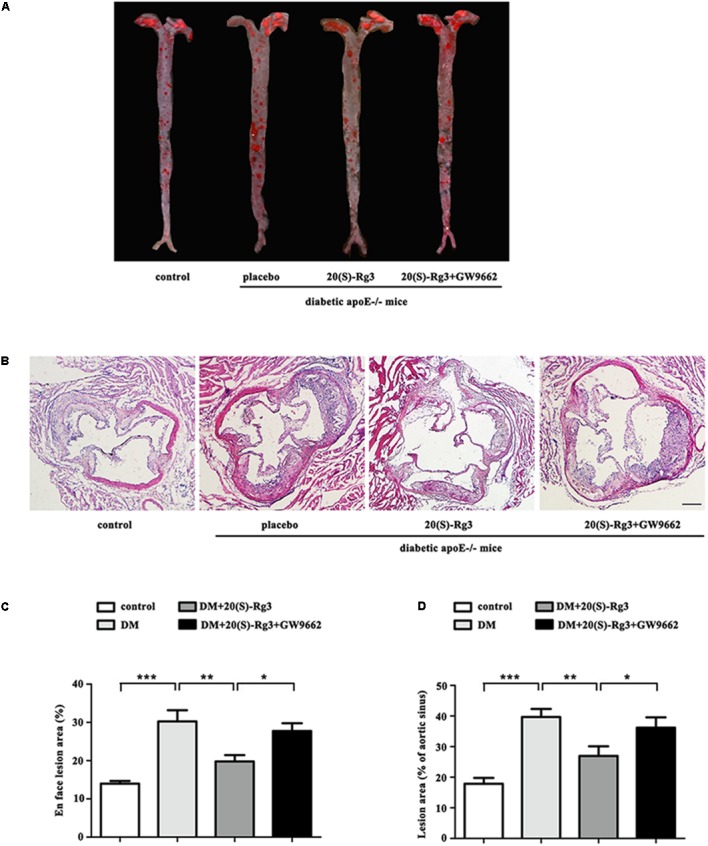
20(S)-Rg3 reduce plaque burden in diabetic apoE–/– mice. **(A)** Representative images of en face Oil-Red O staining of aortas in four groups of mice. **(B)** Quantitative analysis of en face aorta lesions expressed as percentage lesion area relative to total aorta area (*n* = 5, respectively). **(C)** Representative images of cross-sectional aortic root lesions by H&E staining. Scale bar: 200 μm. **(D)** Quantitative analysis of cross-sectional plaque area in aortic roots (*n* = 5, respectively). Data are mean ± SEM. ^∗^*p* < 0.05, ^∗∗^*p* < 0.01, ^∗∗∗^*p* < 0.001.

**FIGURE 5:**
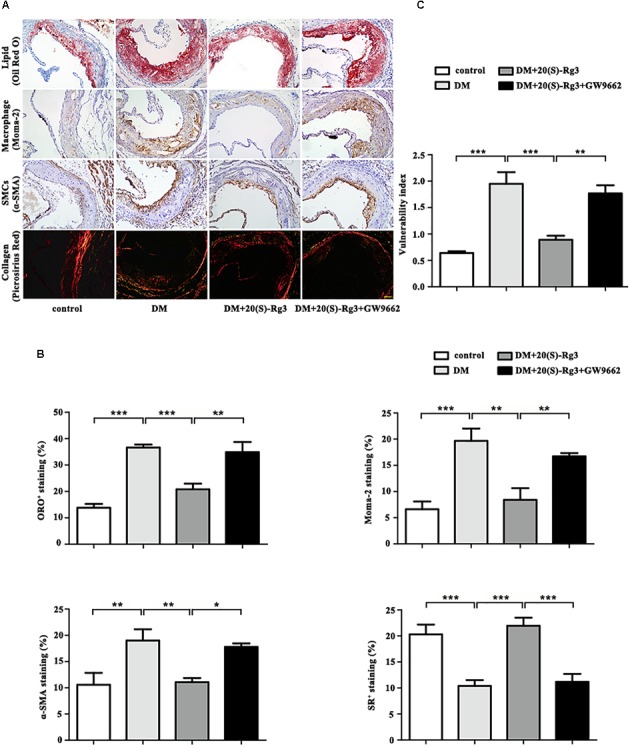
20(S)-Rg3 stabilizes atherosclerotic plaque in diabetic apoE–/– mice. **(A)** Representative staining of lipids, macrophages, smooth muscle cells (SMCs), and collagen in the aortic roots in four groups of mice. **(B)** Quantification of the plaque composition in four groups of mice, expressed as the percentage of the positive staining area to the total plaque area. **(C)** Vulnerability index in four groups of mice (*n* = 5, respectively). Scale bar: 50 μm. Data are mean ± SEM. ^∗^*p* < 0.05, ^∗∗^*p* < 0.01, ^∗∗∗^*p* < 0.001.

As shown in **Figure 4**, the cross-sectional plaque area of the aortic sinus (**Figures 4A,C**) and the relative en face lesion area of the entire aorta (**Figures 4B,D**) were significantly reduced in the 20(S)-Rg3 treated group. Additionally, 20(S)-Rg3 significantly reduced the intraplaque content of lipids and macrophages but increased that of collagen relative to the control group (**Figures 5A,B**). Although there was a decrease in the content of VSMCs, which might contribute to plaque instability, the plaque vulnerability index was actually decreased (**Figure 5C**). GW9662 co-administration mostly reversed the effects of 20(S)-Rg3, indicating that 20(S)-Rg3 induced a more stable plaque composition via PPARγ activation.

### 20(S)-Rg3 Administration Skewed the Macrophage Phenotype Toward Alternatively Activated Macrophages (M2) *in Vivo*

**FIGURE 6:**
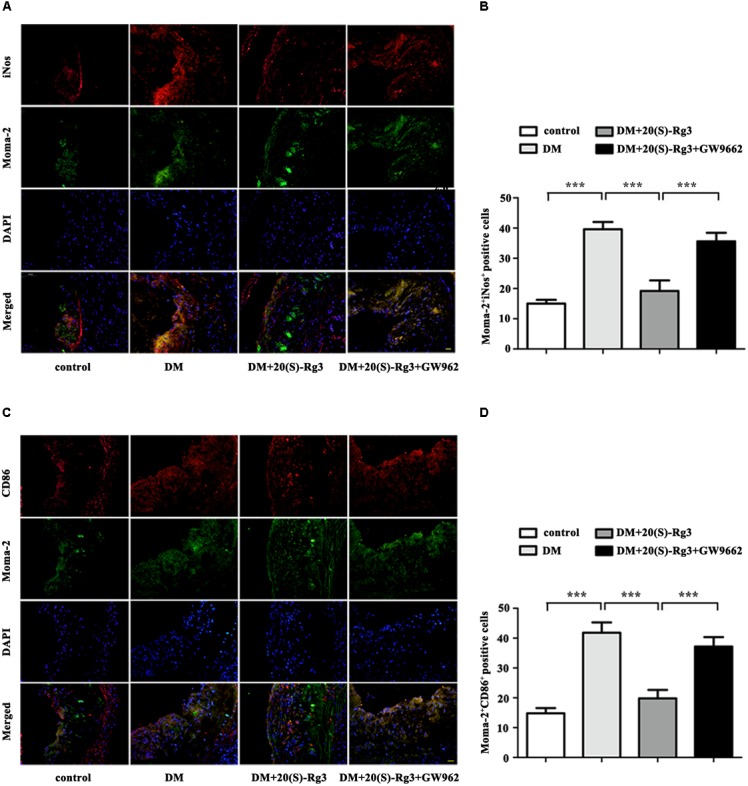
Effects of 20(S)-Rg3 on macrophage polarization in atherosclerotic lesions of diabetic ApoE–/– mice. **(A,B)** Co-immunofluorescence staining of the aortic root for macrophage (anti-Moma-2 antibody, green) and M1 marker iNos (red), and a bar graph summarizing the results (*n* = 5, respectively). **(C,D)** Co-immunofluorescence staining for macrophage (anti-Moma-2 antibody, green) and M1 marker CD86 (red), and a bar graph summarizing the results (*n* = 5, respectively). Scale bar: 20 μm. Data are mean ± SEM. ^∗^*p* < 0.05, ^∗∗^*p* < 0.01, ^∗∗∗^*p* < 0.001.

**FIGURE 7:**
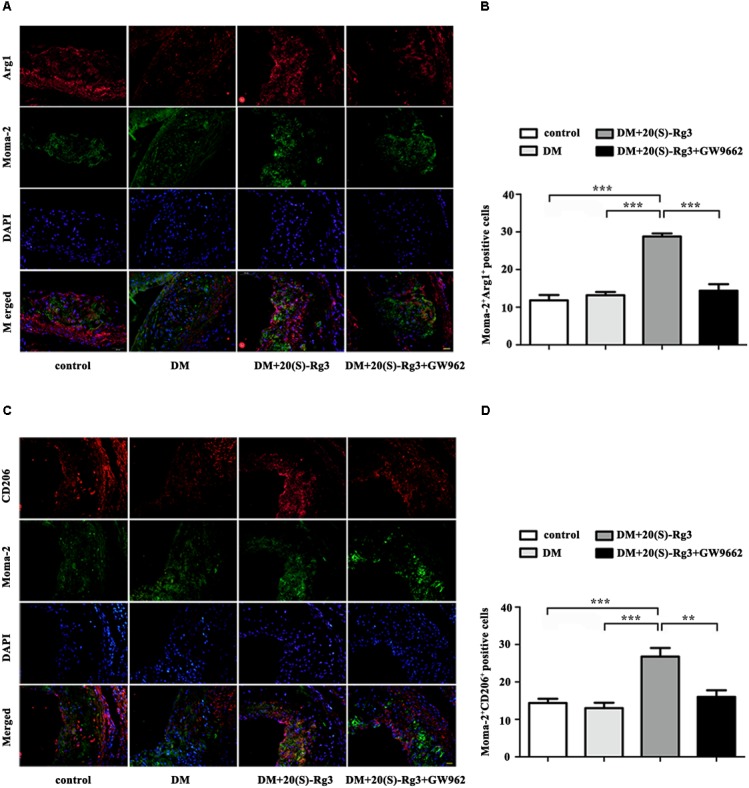
Effects of 20(S)-Rg3 on macrophage polarization in atherosclerotic lesions of diabetic ApoE–/– mice. **(A,B)** Co-immunofluorescence staining of the aortic root for macrophage (anti-Moma-2 antibody, green) and M2 marker Arg1 (red), and a bar graph summarizing the results (*n* = 5, respectively). **(C,D)** Co-immunofluorescence staining for macrophage (anti-Moma-2 antibody, green) and M2 marker CD206 (red), and a bar graph summarizing the results (*n* = 5, respectively). Scale bar: 20 μm. Data are mean ± SEM. ^∗^*p* < 0.05, ^∗∗^*p* < 0.01, ^∗∗∗^*p* < 0.001.

To explore if 20(S)-Rg3 could promote favorable M1/M2 polarization within plaques, we applied double-immunofluorescence to label M1 (Moma-2+iNOS+ or Moma-2+CD86+ double positive) and M2 (Moma-2+Arg-1+ or Moma-2+CD206+ double positive) macrophages. As shown in **Figure 6**, there was minimal detection of M1 macrophages in the control group but robust expression in the DM group. However, after 20(S)-Rg3 treatment, the number of M1 macrophages significantly decreased (**Figures 6A–D**). Conversely, the number of M2 macrophages was minimal in the control and DM groups. In the 20(S)-Rg3 group, the number of M2 macrophages was significantly increased, although the total number of macrophages was reduced (**Figures 7A–D**). These effects of 20(S)-Rg3 were mostly reversed by GW9662 co-administration. Taken together, these results indicate that 20(S)-Rg3 attenuated the infiltration of macrophages and induced macrophage M2 polarization from the M1 sub-type in plaque lesions of diabetic mice.

## Discussion

Inflammation appears to be a common link between atherosclerosis and diabetes, in which activated macrophages play an important role. Both T1DM and T2DM in humans and beta cell destruction/diabetes in rodents result in an increased inflammatory phenotype of monocytes and macrophages (Padmos et al., 2008; Bradshaw et al., 2009; Calle and Fernandez, 2012; Kanter and Bornfeldt, 2013). Indeed, a pathologic assessment of atherosclerotic plaques showed that diabete is associated with more macrophages and lipid-rich areas (Burke et al., 2004), features of unstable plaques. Another study in mice moedel also showed that even in the absence of hyperlipidemia, many of the benefits on plaque composition (fewer macrophages, more collagen) and macrophage phenotype (less inflammation, more M2-like characteristics) were attenuated in diabetes (Parathath et al., 2011). Therefore, strategies trying to suppress hyperglycemia-induced M1 macrophage activation and promote M2 macrophage polarization may be available to stabilize plaques and limit AS progression in diabetes (Wolfs et al., 2011; Liu and Yang, 2013).

**FIGURE 8:**
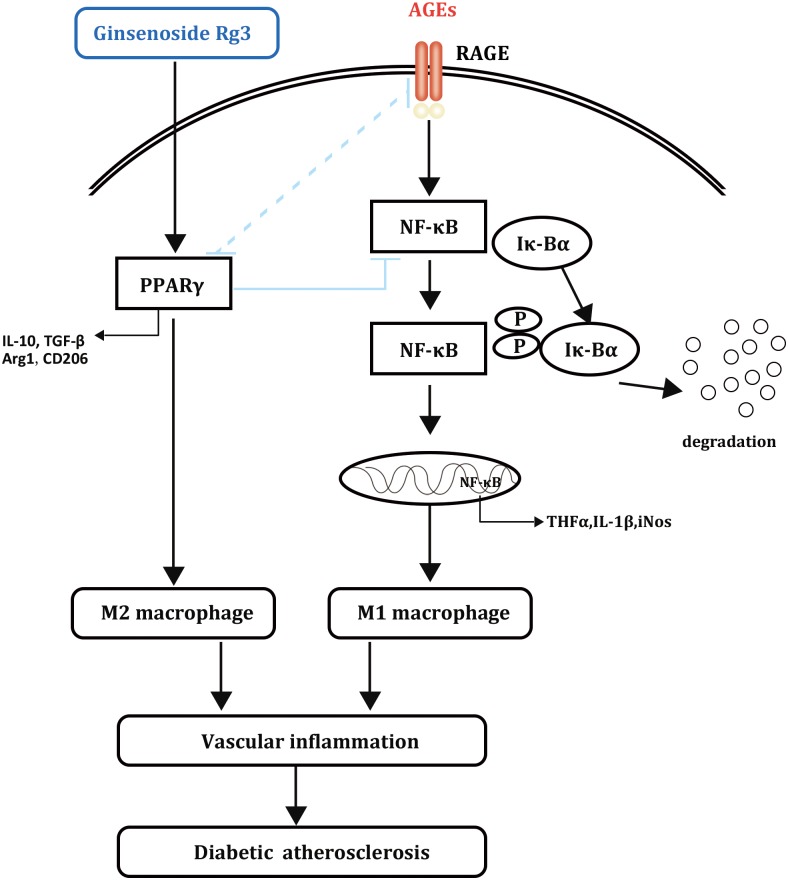
Schematic diagram of the effects of 20(S)-Rg3 on AGEs-induced macrophages polarization.

Ginsenosides such as Rd and Rb1 have been reported to affect macrophage polarization and atherosclerosis progression; however, there has been no related research on diabetic atherosclerosis (Ren et al., 2016; Zhang et al., 2017). This report revealed that 20(S)-Rg3 could reduce the plaque burden and enhance plaque stability in diabetic mice, the effects of which were mostly achieved by suppressing AGEs-induced M1 macrophage activation and promoting M2 macrophage polarization via PPARγ activation (**Figure 8**). Previous studies reported that PPARγ agonists could inhibit the formation of AGEs (Rahbar et al., 2000; Sobal et al., 2005). However, a recent study showed that pioglitazone could inhibit AGEs-induced macrophage M1 polarization via the inactivation of NF-κB, without affecting the level of plasma AGEs or the abundance of AGEs in the aortic plaques (Jin et al., 2016). On the other hand, RAGE, which plays important roles in AGEs-induced macrophage activation, might be downregulated by PPARγ activation in various cells (Zhang Y. et al., 2010; Di et al., 2015). Therefore, the underlying mechanisms by which PPARγ activation modulates macrophage polarization remain to be explored.

In the atherosclerotic lesions from the 20(S)-Rg3 treatment group, we observed a reduction in the plaque burden and an enhancement in plaque stability. 20(S)-Rg3 treatment markedly attenuated lipid accumulation and macrophage infiltration, and increased collagen production. Although there was a decrease in the content of VSMCs, which might contribute to plaque instability, the plaque vulnerability index was actually reduced. Previous studies already implied that PPARγ agonists could inhibit AGEs-induced VSMCs proliferation, an essential event in the development of diabetic atherosclerosis (Wang et al., 2006; Yuan et al., 2011). In the present study, reduced VSMC frequency might come at the price of impaired plaque stability; on the other hand, it contributed to smaller plaque size and reduced plaque burden (Shahzad et al., 2011; Zhou et al., 2015).

To explore the mechanism by which 20(S)-Rg3 protected against atherosclerosis, we investigated the M1/M2 macrophage polarization in atherosclerotic lesions. As expected, 20(S)-Rg3 reduced M1 macrophages (MOMA-2+iNos+/MOMA-2+CD86+) and increased M2 macrophages (MOMA-2+Arg1+/MOMA-2+CD206+) in the plaques of diabetic mice. In addition to regulating the macrophage phenotype in plaques, PPARγ agonists have been reported to decrease the higher proportion of peripheral M1 monocytes/macrophages and improve the M1/M2 imbalance in diabetic circumferences (Satoh et al., 2010; Jin et al., 2016). However, a previous report demonstrated that the M2 to M1 switch during atherosclerosis progression could be due to a phenotypic switch of cells already present in the lesion, but not a recent recruitment of M1 macrophages (Khallou-Laschet et al., 2010). In addition, atherosclerosis and macrophage polarization are also affected by lipid profiles and glucose metabolism (Parathath et al., 2011; Fadini et al., 2014). In our work, 20(S)-Rg3 treatment significantly decreased the levels of blood glucose but not any lipids; therefore, the hypoglycaemic effects might also contribute to its anti-atherosclerotic effects (Tiyerili et al., 2013).

Taken together, ginsenoside 20(S)-Rg3 protected against atherosclerosis by reversing the M1 polarization to the M2 phenotype and limiting intraplaque inflammatory response, which was achieved by PPARγ. Our study provides new experimental evidence for the possibility of 20(S)-Rg3 in the prevention and treatment of atherosclerosis.

## Author Contributions

XJ conceived and designed the experiments. MG, JX, XS, XZ, and YT performed the experiments. MG and JX researched data and contributed to discussion. XS, XZ, LW, and LZ analyzed the data. MG and YT contributed reagents/materials/analysis tools. MG wrote the manuscript. XJ reviewed/edited the manuscript.

## Conflict of Interest Statement

The authors declare that the research was conducted in the absence of any commercial or financial relationships that could be construed as a potential conflict of interest.

## Supplementary Material

FIGURE S1 Cytotoxicity of 20(S)-Rg3. BMDMs and THP-1 cells were treated with indicated concentrations of 20(S)-Rg3 for 24 h. The cell viability was analyzed using CCK8 assay (*n* = 3, respectively). Data are mean ± SEM.
