# Supplementary figures and images for: Ginsenoside Rg3 Mitigates Atherosclerosis Progression in Diabetic apoE–/– Mice by Skewing Macrophages to the M2 Phenotype
Source: Front Pharmacol. 2018 May 9;9:464. doi: 10.3389/fphar.2018.00464 (PMC5954105; doi:10.3389/fphar.2018.00464)

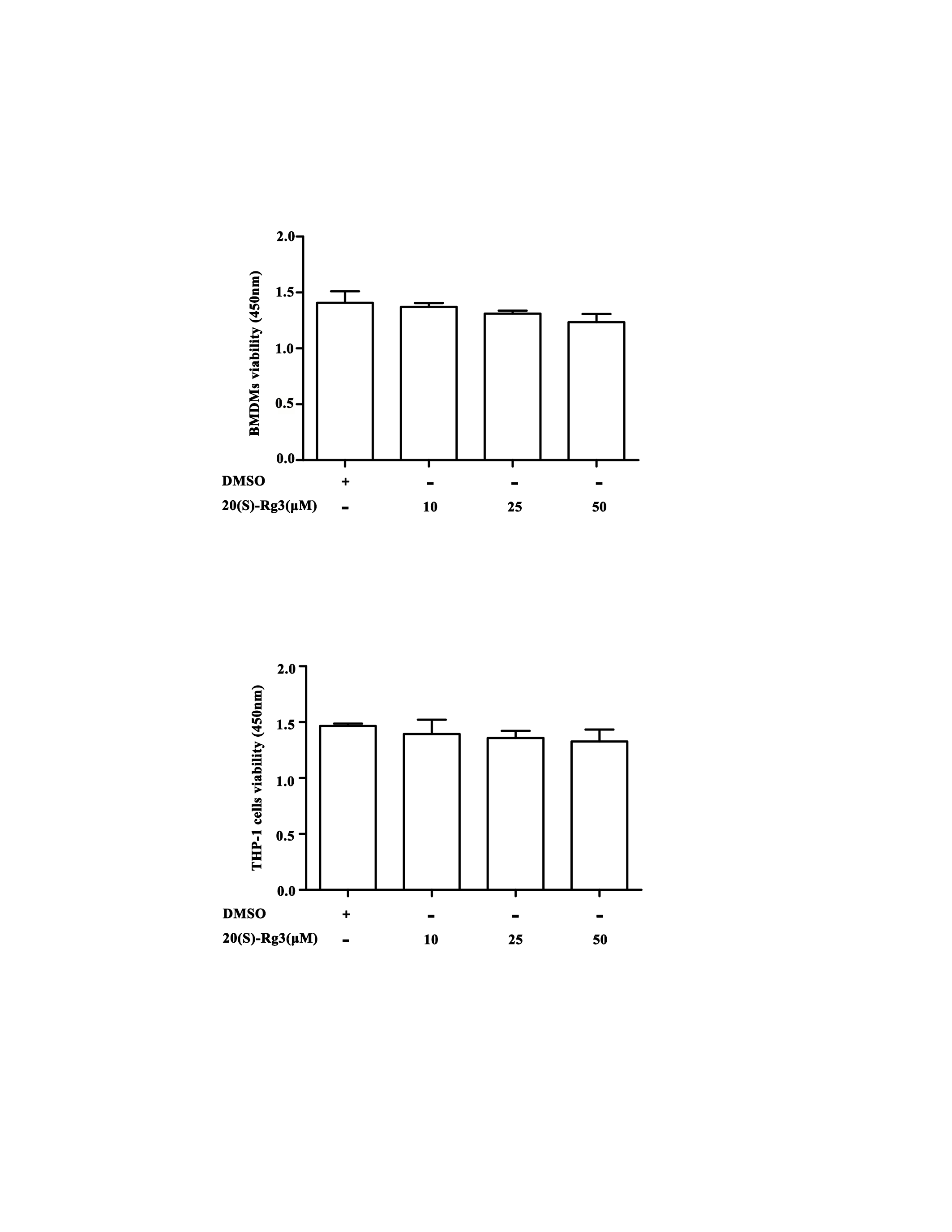

Supplement: FIGURE S1 — Cytotoxicity of 20(S)-Rg3. BMDMs and THP-1 cells were treated with indicated concentrations of 20(S)-Rg3 for 24 h. The cell viability was analyzed using CCK8 assay (n = 3, respectively). Data are mean ± SEM. [file Image_1.TIF]
